# Determinants of death among tuberculosis patients in a semi urban diagnostic and treatment centre of Bafoussam, West Cameroon: a retrospective case-control study

**DOI:** 10.11604/pamj.2015.22.253.6576

**Published:** 2015-11-18

**Authors:** Fabrice Nembot Djouma, Michel Noubom, Armelle Viviane Ngomba, Hubert Donfack, Patrick Stéphane Mfin Kouomboua, Michael Amede Fopa Saah

**Affiliations:** 1 Department of Biomedical Sciences, University of Dschang, Cameroon, Dschang, Cameroon; 2 Department of Biomedical Sciences, University of Dschang, Dschang, Diagnostic and Treatment Centre of Baleng, Bafoussam, Cameroon; 3 Central Technical Group Expanded Program on Immunization, Ministry of Public Health, Yaounde, Cameroon

**Keywords:** Tuberculosis, death, factors, Cameroon

## Abstract

**Introduction:**

Tuberculosis (TB) remains a worldwide public health problem with 8.6 millions of new cases and 1.3 millions of death annually. Despite the progress recorded in fighting against this disease in the recent years, Africa is still not on the track to achieve the objective to reduce by half the death rate due to this disease by 2015.

**Methods:**

A case-control study was conducted on data of patients admitted for tuberculosis between 1996 and 2011 in the Diagnostic and Treatment Center of Baleng. Cases were patients who died from any cause during anti tuberculosis treatment. Logistic regression model was used to identify factors associated to death.

**Results:**

In 4201 patients treated during the study period, 3245 (77.24%) were included in the study. The mean age was 35.9 (SD 14.2) and male represent 62.2% (CI 60.6- 63.9) of them. At the end of the follow up, 2883 patients were successfully treated, 362 died during treatment and 132 (36.5%) deaths occurred during the first two months of TB treatment. HIV positive status, Extra-pulmonary TB, sputum smear-negative pulmonary TB and male sex were significant independent risk factors of death with adjusted odds ratio of 4.8 (CI 3.2- 7.4); 3.0 (CI 1.6- 5.4); 2.7 (CI 1.7- 4.4) and 1.5 (CI 1.0- 2.3) respectively.

**Conclusion:**

The mortality rate of TB patients undergoing TB treatment remains high. Studies are needed to identify and test efficient interventions of mortality reduction among TB patients in resource limiting settings.

## Introduction

With 8.7 million new cases and a specific mortality of 1.4 million cases of death in 2011, tuberculosis remains a major public health problem in the world [1]. In 2012, African region was ranked the second region with of the highest TB cases with about 27% of worldwide cases [1]. In the same year, the prevalence of TB cases and death in Cameroon was estimated at 69 thousands and 14.1 thousands respectively [2]. The fight against TB remains a worldwide challenge. It was reinvigorated in 1991, when a World Health Assembly (WHA) resolution recognized TB as a major global public health problem [3]. From that time, all actions in TB control aimed to reduce by 2015 the TB prevalence and death rates by 50% relative to 1990 [4]. In the 18 years since the launch of a new international strategy for TB care and control by WHO in the mid-1990s (the DOTS strategy) and the subsequent global rollout of DOTS and its successor (the Stop TB Strategy) [5], major progress has been observed. Globally, the TB mortality rate has fallen by 45% since 1990 (with about 4.66.3 million lives saved). It has also been observed that the global TB incidence rate is falling in most parts of the world [6]. Despite these progresses, TB remains the second leading cause of death from an infectious disease worldwide [1]. The TB specific rate deaths is unacceptably high given that most of cases are preventable if patients can access health care for a diagnosis and if they are adequately treated [1]. According to WHO, additional efforts are needed in African Region which is currently not on track to achieve the mortality and prevalence targets [1]. These efforts should include the identification of the determinants of death among patients undergoing anti tuberculosis treatment. Factors associated to death among TB patients are worldwide documented. They are HIV positive status, immune depression, negative pulmonary TB, malnutrition, co-morbidity with non-infectious diseases, abusive alcohol and drug used [7]. In Cameroon, the literature describes characteristics of patients who died during tuberculosis treatment [8]. More information is needed to improve the survival of TB patients treated on Cameroonian Anti-tuberculosis Program. In the process of identifying interventions to reduce tuberculosis burden, there is a need to identify factors associated to death among TB patients. This study had as objective to identify, amongst patients diagnosed with TB between 1996 and 2011in Baleng TB Diagnostic and Treatment Center (DTC) of West Cameroon, factors associated to TB death.

## Methods

### Study design

It was retrospective case-control study. Cases were defined as patients who died from any cause during anti tuberculosis treatment and controls were defined as patients successfully treated. Data collection was done on medical records of TB patients who came to DTC of Baleng between 1996 and 2011.

### Study site

The DTC of Baleng is the largest DTC in the western region of Cameroon with more than 20 beds exclusively for tuberculosis patients. It is located in a semi urban town belonging in Mifi Health District. In DTC of Baleng, patients are treated according to the WHO Direct Observed Treatment Strategy (DOTS) [9]. At the end of patients’ follow up, they are classified into six groups[1]: cured (patients who are initially sputum smear-positive and who are sputum smear-negative in the last month of treatment and on at least one previous occasion); Completed treatment (patients who complete treatment but do not meet the criteria for cure or failure); Failed (patients who are initially sputum smear-positive and who remain sputum smear-positive at month 5 or later); Defaulted (patient whose treatment are interrupted for two consecutive months or more); Transferred (patients who are transferred through another DTC during anti tuberculosis treatment) and Died (patients who die from any cause during treatment). The patients who are cured or those who completed treatment are grouped into successfully treated.

### Data management

Data was collected from April to august, 2012 by trained surveyors. They focused on patients’ socio demographic characteristics, previous contact with anti tuberculosis treatment, HIV status, and clinical files of tuberculosis and treatment outcome. Epi info software version 3.5.3 was used for data entry and data analysis. In general analysis, proportions (with 95% Confident Interval: 95% CI) and means (with Standard Deviation: SD) were calculated. During case-control analysis, patients with unknown mortality status (referred and those who were lost to follow up) were excluded. Patients with incomplete data regarding exposure to suspected risk factors were also excluded. For univariate analysis Chi squared test (for qualitative variables) and Student's T-test (for quantitative variable) were used. To adjust the estimation of Odds Ratio (OR), multivariate logistic regression models were constructed. For the final model, significant predictors were selected using forward stepwise methods. P-values under 0.05 were considered as significant. As data were collected in patient's register, no informed consent was necessary. The study was approved by the administrative authorities of the Diagnostic and Treatment Center of Baleng and Mifi Health Committee.

## Results

### General descriptive of participants

**Figure 1:**
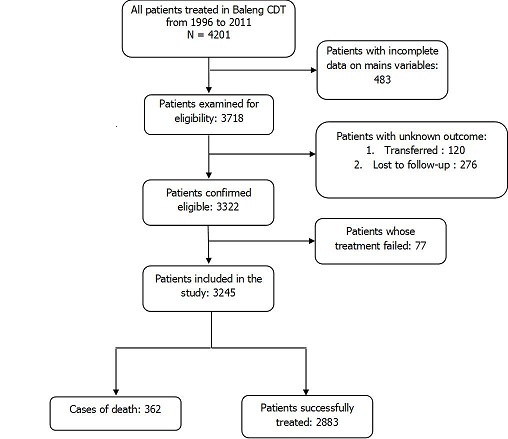
Participants included in the study

**Figure 2:**
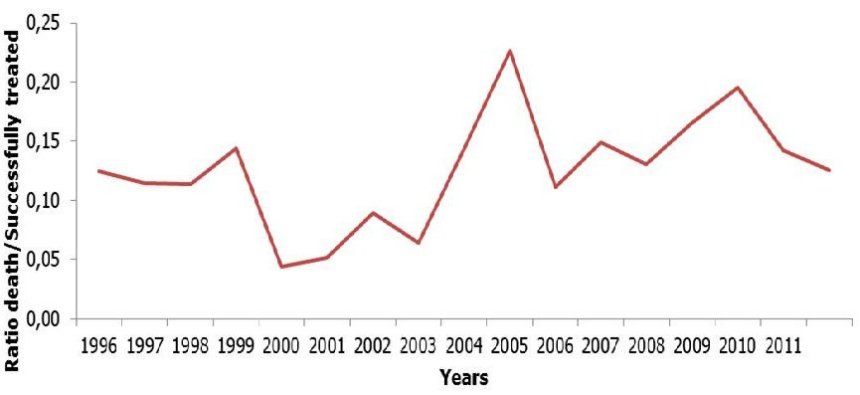
Evolution of Ratio death/successfully treated in diagnostic and treatment center of Baleng

In this study, 362 cases of death and 2883 patients successfully treated were included in analysis as presented in Figure 1. The mean age at the time of diagnosis was 35.9 (SD 14.2) years old and male sex was predominant with 2020 participants (62.2% CI 60.6- 63.9). Age was further categorized into two groups: patients with age less than or equal to 40 years old and those with age above 40 years old. The number of participants aged more than 40 was 969 (29.9% CI 28.3-31.5). Among patients included in analysis, 2885 (90.9- 92.8) were new cases of TB whereas 255 (81% CI 7.2- 9.1) had an antecedent of anti-tuberculosis treatment. Clinical forms of TB were: Extra-pulmonary Tuberculosis, sputum smear-positive pulmonary TB and sputum smear-negative pulmonary TB with respective frequencies of 145 (4.5% CI 3.8- 5.3); 2677 (82.4% CI 78.4- 86.2) and 423 (13.0% CI 8.9- 17.1). Among participants with known HIV status (35.1%), 305 (26.9% CI 24.3- 29.6) were positive and among them, 77 (25.2% CI 19.9- 30.5) had been documented as taking antiretroviral therapy (ART). Death occurred for 132 (36.5%) patients before the end of the first two months of intensive phase of treatment. Globally, the ratio death/successfully treated was 0.13 and his evolution by years is presented in Figure 2.

### Factors associated to death

In univariate analysis, as presented in Table 1, socio demographic-related risk factor which was significantly associated (p < 0.05) with death was age > 40 years. Clinical-related risk factors which were significantly associated with death were Extra-pulmonary localization of TB and Sputum smear-negative pulmonary. Male sex and antecedent of anti TB treatment were associated to patients’ death but not significantly with p value of 0.31 and 0.21 respectively. Significant independent risk factors for death in multivariable logistic regression model were male sex, Sputum smear-negative pulmonary TB, extra pulmonary TB and HIV positive status. Details of multinomial logistic regression model are presented in Table 2.

**Table 1:** Comparison of death and successfully treated groups according to socio demographic and clinical characteristics

Characteristics	Frequencies		OR (95%CI)	p value
	Death cases (N = 362) (n,%)	Successfully treated (N= 2883) (n,%)		
Male sex	234 (11.6)	1786 (88.4)	1.1 (0.8- 1.4)	0.31
Age > 40 years	115 (16.0)	814 (84.0)	1.9 (1.5- 2.3)	< 0.01
Extra pulmonary TB	51 (35.2)	94 (64.8)	4.8 (3.3- 6.9)	< 0.01
Sputum smear-negative pulmonary TB	86 (20.3)	337 (79.7)	2.7 (2.1- 3.6)	< 0.01
Antecedent of anti TB treatment	32 (12.5)	223 (87.5)	1.2 (0.8- 1.7)	0.21
HIV positive	78 (25,6)	227 (74,4)	4,5 (3,1- 6,6)	< 0,01
Anti-Retroviral Treatment	17 (22,1)	60 (77,9)	0,7 (0,4- 1,3)	0,14

**Table 2:** Multinomial logistic regression of factors associated to death of TB patients treated in DTC of Baleng, 1996- 2011

Characteristics	Adjusted OR	95% Confidence Interval	p value
Male sex	1.5	1.0- 2.3	0.04
Age > 40 years	1.3	0.9- 2.1	0.13
Sputum smear-negative pulmonary TB	2.7	1.7- 4.4	<0.01
Extra pulmonary tuberculosis	3.0	1.6- 5.4	<0.01
HIV positive	4.8	3.2- 7.4	<0.01

## Discussion

This study which aimed to identify factors associated to TB patients’ death has shown that, male sex, sputum smear-negative pulmonary TB, Extra-pulmonary TB and HIV positive status were significant independent risk factors for death among TB patients treated in DTC of Baleng between 1996 and 2011. During the study, data were extracted from TB patients’ records which can be a source of potential information biases. Despite it, the power of the study was good due to the number of participants (2883) and to the number of deaths (362). The strongest factor associated to death among study population was the positive HIV status. This association has been observed in others studies implemented in different settings [7, 10–12]. The synergy action of M. tuberculosis and HIV is well known and documented. These two pathogens potentiate one another, accelerating the deterioration of immunological functions and resulting in premature death if untreated or inadequately treated [13]. Taking into account the high burden of these diseases and their effects on mortality of co-infected patients, the World Health Organization recommend implementation of collaborative TB/HIV activities. One of these activities is the provision of antiretroviral therapy (ART) to all TB patients with HIV infection [4]. The effect of ART on the survival of TB patients is well documented [14–16]. Despite this benefit of ART, the implementation of collaborative TB/HIV activities on the field remains difficult due to several management challenges that compromise programmatic implementation in resource-limited settings [17]. In our study for example, only 25.2% (77) of patients with HIV positive status were on ART. Despite it, ART was protective factor of death in univariate analysis. The second factor identified as associated to TB patients’ death in our study was Extra-pulmonary localization of TB. In fact, patients with Extra-pulmonary localization of TB had three times more risk to die compared to those with pulmonary TB. Extra-pulmonary TB as factor associated to death has been documented in Barcelona, Spain after a population-based cohort study [12]. On the other hand, analysis of European surveillance data has shown that Extra-pulmonary TB is protective of death compared to pulmonary TB. Explanation which can be given to this is the rarity of severe forms of Extra-pulmonary TB in the European population. Another explanation can be the fact that during that European study, patients with both pulmonary and Extra-pulmonary TB were included under pulmonary TB [18]. Nevertheless, at our knowledge, evaluation of association between Extra-pulmonary TB and patients’ death is not yet explored in sub-Saharan African Countries. Further study should therefore focus on the exploration of that association. The third strongest factor associated to death was sputum smear-negative disease. That association has been shown by other studies elsewhere [7, 10]. This association can be explain by the fact that, in setting with high prevalence of HIV, sputum smear-negative is the expression of advance immune depression [7, 19]. And thus, the deaths in these cases are due to opportunistic diseases related to AIDS. In our study, the male sex was also associated to patients’ death. The same results have been shown by others studies [11, 18, 20]. However, tuberculosis among women remains a major public health issue because it is among the top three killers of women worldwide [1]. WHO estimate that, in most low-income countries, twice as many men are notified with tuberculosis as women due to socioeconomic and cultural factors leading to barriers in assessing health by women [21]. Intervention aimed to reduce mortality among TB patients should thus target both men and women.

## Conclusion

The aim of this study was to identify factors associated to death among tuberculosis patients treated under Cameroonian National Anti-tuberculosis Program. After the reviewed of TB patients’ records treated from 1996 to 2011 in a major DTC of Bafoussam, West Cameroon, factors associated to patients’ death was HIV positive status, Extra-pulmonary TB, sputum smear-negative pulmonary TB and male sex. These groups at risk have to be closely follow-up during anti-tuberculosis treatment to achieve the sixth Millennium Development Goals. Moreover, studies are needed to identify and test efficient interventions of mortality reduction among TB patients in limited settings.

## References

[1] World Health Organisation (2013). Global Tuberculosis Report 2013.

[2] World Health Organisation Cameroon country profile.

[3] Resolution WHA44.8 (1993). Tuberculosis control programme. Handbook of resolutions and decisions of the World Health Assembly and the Executive Board. Volume III, 3rd ed. (1985-1992).

[4] World Health Organization: THE STOP TB STRATEGY: Building on and enhancing DOTS to meet the TB-related Millennium Development Goals (2006).

[5] Raviglione M, Uplekar M (2006). WHO’s new Stop TB strategy. Lancet.

[6] Glaziou Philippe, Floyd Katherine, Korenromp Eline, Sismanidis Charalambos, Bierrenbach Ana, Williams Brian, Atun Rifat, Raviglione Mario (2011). Lives saved by tuberculosis control and prospects for achieving the 2015 global target for reducing tuberculosis mortality. Bull World Health Organ..

[7] Waitt CJ, Squire SB (2011). A systematic review of risk factors for death in adults during and after tuberculosis treatment. Int J Tuberc Lung Dis..

[8] Kuaban C, Koulla-Shiro S, Hagbe P (1997). Caractéristiques des patients adultes morts de tuberculose pulmonaire active à Yaoundé-Cameroun. Médecine d'Afrique Noire..

[9] World Health Organization Global tuberculosis control: WHO report 2011.

[10] Lawn SD, Acheampong JW (1999). Pulmonary tuberculosis in adults: factors associated with mortality at a Ghanaian teaching hospital. West Afr J Med..

[11] Horne David, Hubbard Rebecca, Narita Masahiro, Exarchos Alexia, Park David, Goss Christopher (2010). Factors associated with mortality in patients with tuberculosis. BMC Infect Dis..

[12] Millet Juan-Pablo, Orcau Angels, Rius Cristina, Casals Marti, Olalla Patricia Garcia (2011). Predictors of Death among Patients Who Completed Tuberculosis Treatment A Population-Based Cohort Study. PLoS ONE..

[13] Pawlowski Andrzej, Jansson Marianne, Skoïld Markus, Rottenberg Martin, Kaïllenius Gunilla (2012). Tuberculosis and HIV Co-Infection. PLoS Pathog..

[14] Schmaltz Carolina Arana Stanis, Santoro-Lopes Guilherme, Lourenço Maria Cristina, Morgado Mariza GonÇalves, Velasque Luciane de Souza, Rolla Valéria Cavalcanti (2012). Factors Impacting Early Mortality in Tuberculosis/HIV Patients: Differences between Subjects Naïve to and Previously Started on HAART. PLoS ONE..

[15] Karim Salim Abdool, Naidoo Kogieleum, Grobler Anneke, Padayatchi Nesri, Baxter Cheryl, Gray Andrew (2010). Timing of Initiation of Antiretroviral Drugs during Tuberculosis Therapy. N Engl J Med..

[16] Manosuthi Weerawat, Chottanapand Suthat, Thongyen Supeda, Chaovavanich Achara, Sungkanuparph Somnuek (2006). Survival Rate and Risk Factors of Mortality Among HIV/Tuberculosis-Coinfected Patients With and Without Antiretroviral Therapy. J Acquir Immune Defic Syndr..

[17] Cohen K, Meintjes G (2010). Management of individuals requiring ART and TB treatment. Curr Opin HIV AIDS..

[18] Lefebvre N, Falzon D (2008). Risk factors for death among tuberculosis cases: analysis of European surveillance data. Eur Respir J..

[19] Kingkaew N, Sangtong B, Amnuaiphon W, Jongpaibulpatana J, Mankatittham W, Akksilp S, Sirinak C, Nateniyom S, Burapat C, Kittikraisak W, Monkongdee P, Varma JK (2009). HIV-associated extrapulmonary tuberculosis in Thailand: epidemiology and risk factors for death. Int J Infect Dis..

[20] Vasankari Tuula, Holmström Pekka, Ollgren Jukka, Liippo Kari, Kokki Maarit, Ruutu Petri (2007). Risk factors for poor tuberculosis treatment outcome in Finland: a cohort study. BMC Public Health..

[21] Connolly M, Nunn P (1996). Women and tuberculosis. World Health Stat Q..

